# Virtual Communities for Diabetes Chronic Disease Healthcare

**DOI:** 10.1155/2011/721654

**Published:** 2011-11-03

**Authors:** Ivan Chorbev, Marija Sotirovska, Dragan Mihajlov

**Affiliations:** Faculty of Computer Science and Engineering, Ss. Cyril and Methodius University in Skopje, P.O. BOX 574, 1000 Skopje, Macedonia

## Abstract

Diabetes is classified as the world's fastest-growing chronic illness that affects millions of people. It is a very serious disease, but the bright side is that it is treatable and can be managed. Proper education in this view is necessary to achieve essential control and prevent the aggregation of this chronic sickness. We have developed a healthcare social network that provides methods for distance learning; opportunities for creation of virtual self-help groups where patients can get information and establish interactions among each other in order to exchange important healthcare-related information; discussion forums; patient-to-healthcare specialist communication. The mission of our virtual community is to increase the independence of people with diabetes, self-management, empower them to take care of themselves, make their everyday activities easier, enrich their medical knowledge, and improve their health condition, make them more productive, and improve their communication with other patients with similar diagnoses. The ultimate goal is to enhance the quality of their life.

## 1. Introduction

### 1.1. Diabetes

Diabetes is a disorder of the human metabolism—the way the body uses digested food for energy or growth. Most of the food people eat is broken down into glucose, the form of sugar in the blood. Glucose is the main source of fuel for the body [1]. Diabetic patients lack the ability to use glucose properly, so in their case it is stored in their bloodstream causing numerous difficulties.

Diabetes is classified as the world's fastest-growing chronic illness that affects millions of people. According to World Diabetes Foundation, the prevalence of diabetes has reached epidemic proportions. An estimated 285 million people, corresponding to 6.4% of the world's adult population, live with diabetes in 2010. The number is expected to grow to 438 million by 2030, corresponding to 7.8% of the adult population [2]. Based on IMG statistics and future projections, in 2000 there were 54 000 people with diabetes in our country, and in 2030 it is expected that the number of people with diabetes will rise to 96 000 [3]. Diabetes affects 25.8 million people of all ages in the USA, 8.3 percent of the US population. Diagnosed are 18.8 million people; undiagnosed are 7.0 million people [4]. The bright side is that diabetes is treatable and can be managed. Diabetic people might live a seemingly normal life, but they need to have a continual treatment and diet which is very important.

Appropriate education in this view is necessary to achieve essential control and prevent the aggregation of the chronic sickness. Healthy eating, physical activity, and blood glucose testing are the basic management tools for diabetes [1]. People with diabetes must take huge commitment for their every day care. It is very important that they should have a health care provider that will help them learn to manage their disease and will monitor their diabetic condition.

### 1.2. Virtual Communities

As Calvin M. L. Chan, Mamata Bhandar, Lih-Bin Oh, Hock-Chuan Chan stated, virtual communities represent “social aggregations of people carrying out public discussion long enough, with sufficient human feeling, to form webs of personal relationships in cyberspace” [5]. Virtual professional communities have become a topic of interest for many researchers in the last few years. In fact, research on the use of social networking applications in various areas has become increasingly popular and fruitful. Mohyuddin, W. A. Gray, David Morrey and Wendy Jones present in their introduction in the Incorporating Wireless Technology into Virtual Organizations Supporting the Work of Healthcare Teams that “…initial analysis suggested that an approach based on creating a virtual organization in the distributed computing environment would enable collaborative working to be supported. The virtual organization would have access to a variety of information resources traditionally used in medical healthcare systems, but would utilize wireless technology to support the point of care activities of the members of the care team” [6]. As virtual communities become more popular, researchers need to be conscious of what is happening in the theoretical dimension as well as practical evolution. Attention should be focused on research opportunities that exist.

### 1.3. Diabetes Healthcare Virtual Communities

One of the advances in diabetes healthcare in the last few years is the widespread helpfulness of an electronic peer-to-peer community, where people with mutual interests come together “virtually” to ensure mutual support and self-help, share experiences, and ask questions. Self-care is a recent initiative in the healthcare industry that targets to treat patients with long-term conditions nearer to home and earlier in the course of their disease [7]. It has received great attention because it is the most common and cost-effective form of treatment of many diseases and provides possibilities of predicting and therefore avoiding serious complications. Wireless data-transfer technologies enable not only constant online health state followup, but also an opportunity for each patient's special treatment and individual advice. Monitoring can be performed anywhere and anytime, without cables' limitations. It is effective and leads to better care and reliability [7]. Access to diabetes disease information is much easier; patients may learn from the real world experiences of other patients with the same medical condition and to share their problems with others. Remote monitoring of chronic disease can lower health care costs and increase patient satisfaction and quality of life [8].

Virtual communities for diabetes healthcare play an important role in contributing to the overall effect of diabetes treatment worldwide. “A key aspect of successful chronic disease management is active partnership between consumer and provider—this is particularly important in diabetes management, where many key activities are in the hands of the patient” [9]. A serious work into combining social networks and healthcare is presented in [10]. An international user group is selected as a test bed for a myriad of provided online social services constrained with the specifics of healthcare. The presented results show a promising future for medical social networks. A similar effort focused on patients using insulin pumps is given in [11].

There are various educational materials and knowledge-based systems, data-mining applications and other analytical systems used to ease learning and improve the learner's knowledge. Still however, the quality of the knowledge and the ways of knowledge transfer from one learning stage to another within an automated learning environment is facing challenges. Also, the learner often has no opportunities to transfer the learned materials to practice [12]. This is an additional reason why social networking is expected to bring fresh advances in medical education. Newly developed software programs and Internet technologies that are in continuous growth are making diabetic patients' coping with the disease easier, considering that they can find useful health information online. Virtual communities can provide communication, collaboration and information collection, and sharing in the diabetes health care community. 

One of the major issues when combining medicine and the Internet is the control of information, its verification, privacy issues, and the possibility of incorrect offers and statements. These kinds of healthcare virtual communities can be very helpful, but robust verification mechanisms are required. In order for this kind of applications to be fully completed, there is a need of specially designed measurement protocols, real-time automatic data processing algorithms, intuitive human interfaces, and secure data transmission over public networks [13].

## 2. Diabetes Healthcare Virtual Community Model

We have developed a system that provides methods for distance teaching and learning; opportunities for creation of self-help groups where patients can get information and establish interactions with other patients in order to enhance their life experience; discussion forums; patients-to-healthcare-specialist communications. The mission of our virtual community is to increase the independence of people with diabetes, empower them to take care of themselves in their everyday activities, enrich their medical knowledge, improve their health status or condition, make them more productive, and facilitate communication. It is all done with one main goal, to enhance the quality of their life.

**Figure 1:**
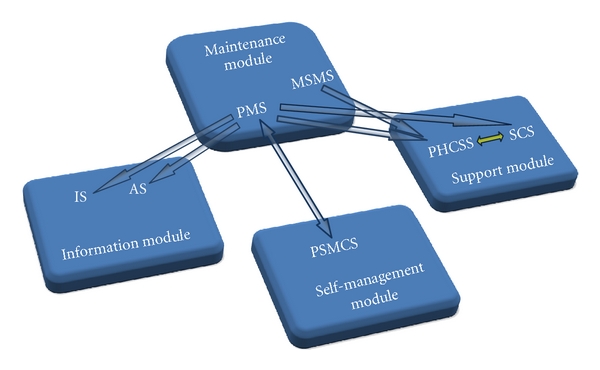
The architecture of Diabetes Mobile Virtual Platform (PMS: Patient Maintenance Service, MSMS: Medical Specialist Maintenance Service, PHCSS: Professional Health Care Support Service, SCS: Social Collaboration Service, PSMCS: Patient Self-Management Care Service, IS: Information Service, AS: Advertising Service).

The benefits from the system should be attractive for both sides: healthcare delivery on one hand and business perspectives so that the system can be supported on the other hand. Being a union of web application modules, mobile device applications, all based on a secure infrastructure, the proposed diabetes virtual model enables healthcare anywhere, at any time, on any device. The system architecture is shown in Figure 1.

The system is developed using Microsoft-based technologies, ASP. NET, and MS SQL Server 2008. The Model View Presenter pattern was used to ensure modularity, fast response times, multilayered architecture, data consistency, and easy redesign of the user interface. AJAX technologies are used to minimise network traffic, speed up the work, and provide a desktop-like user experience.

This model differs in that it offers new ways of delivering e-health services and diabetes disease management. Its platform contains several supporting services.

**(i) Patient Maintenance Service**

This service facilitates registration of new members, people with diabetes, their family members, or anyone else with an interest in the disease. The user only needs to fill the registration form with their private information and choose the account's username and password. Once the information is confirmed and the registration is successful, the user can login and use the opportunities that other services offer. Management of the user's profile entered during the registration is also enabled by this service. The Profile Information Module (personal module that appears when members login) presents member's information like name and surname, phone numbers, email address, birthdays, and so forth. An appropriate security level is established; no one but himself can see the information entered by the member into the Profile Information Module.

**(ii) Medical Specialist Maintenance Service**

In parallel with the Patient Maintenance Service is the Medical Specialist Maintenance Service, manageable by medical specialists. The medical specialists, who are assigned to help members of the mobile virtual community, have the opportunity to change their private information and professional expertise. The attached medical specialist's information enables the patients to make an informed decision on who is the best health care professional to address through the Professional Health Care Support Service for the particular problem they experience.

**(iii) Patient Self-Management Care Service**

This service enables patients to increase their self-management by dealing with their health issues outside of medical institutions. The system offers them appropriate guidance and advice customized according to their specific needs. Each member has an opportunity to update details about their health parameters including blood sugar concentration (blood glucose level is the amount of glucose (sugar) present in the blood of a human), each day or several times per day. Also a measure of the glycosylated hemoglobin-HbA1c can be entered. Blood glucose measurements are presented in the user's blood sugar regulation report. The report gives the patient comparative content per time and offers excellent monitoring and controlling. Regularly updated health parameters enable customized advice and guidance for the patient in question.

**(iv) Social Collaboration Service**

**Figure 2:**
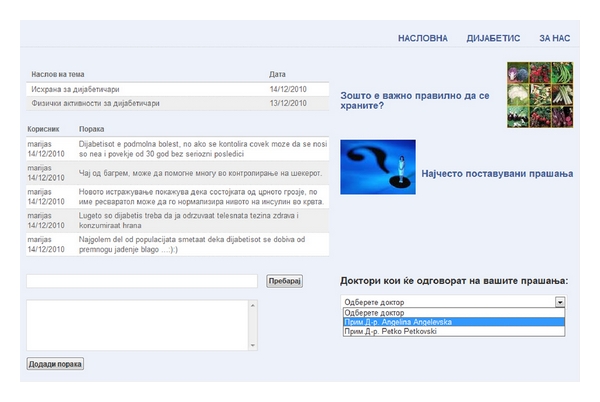
Social collaboration service.

This service facilitates communication among patients. The goal is to exchange experiences and advice. Connections among patients are an important way to receive information and support. An essential component of the social connection through the application is the psychological dimension of sharing the problems among patients. As part of this service, there is an online discussion site, a forum where members can converse by using posted messages. Each member can add new forum topics as issues arise and can also participate in existing forums. The internet-based forums are of even greater importance for patients without access to a vis-a-vis support group in their area. The service is essential to patients that prefer their privacy and would rather communicate and search information online as opposed to visiting support groups or other public institution (Figure 2).

The social module contains the usual befriending mechanisms of social networks, but the differences mainly consist of data security and privacy levels. We defined several levels of friendship (groups in which friends can be classified) sorted by decreasing level of confidence:

1. intimate,
2. close,
3. acquaintances,
4. no trust.

The user can create custom groups and associate friends in the groups. When materials are published by the user, they choose which group of friends can access them. Doctors are placed in parallel groups:

1. personal physician,
2. temporary advice.

This service is directly connected with the Professional Health Care Support Service by implementation of security and privacy policies that are set by the patients themselves. When patients need professional health care attention, they send an email to the desired specialist. At the same time the patient can choose whether the message will be public or private. If the message is made public, it can be easily transformed into a forum topic. In this case the specialist's answer is also public and made part of the newly created forum. The communication is visible to all other members, not only to the sender himself. The feature helps people that have common interests, problems, or goals. Certainly, a patient may decide to send private questions to the specialists. In this case data security and trust are guaranteed.

As in any social network, privacy of published data is essential. In the medical network issues of privacy are of even greater importance. The system allows creation of users with hidden identities, as well as public. Each published content can be controlled in terms of who can access it. Access control is based on groups of users as well as inclusion or exclusion of specific users for specific posted information (Photo, Lab results, Narrative postings).

Unlike popular social networks, the terms of use here state that after a removal of the posted content by the patient/user, the content is permanently erased. The data remains solely the property of the user. Special permission can be granted by the user to allow the use of some of their data in data mining and statistical analysis for medical purposes. A notification is sent by the administrator to ask for granting permission for a specific piece of information, naturally always stripped of any identification when used later in research.

**(v) Professional Health Care Support Service**

**Figure 3:**
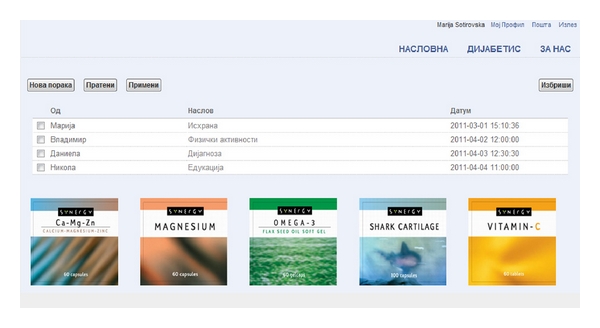
Professional health care support service.

Professional support is essential for the patient. Psychological help for diabetes patients is also very important. Knowing that there is always someone that can help dealing with the pressure and stress involved in daily life situations is very relievable for patients. Advisors are available to assist members by viewing their health history and by answering their questions regarding their health condition. Also, they offer email notifications for appointments, services for rescheduling or canceling the appointments, services for requesting prescriptions, and access to other health resources (Figure 3).

**(vi) Information Service**

This service provides members with necessary information about their disease. It is very important that patients know as much as possible for their disease. Knowledgeable patients tend to adapt easier to effective measures of disease control and prevent complications. The frequently asked questions module offers answers to various questions patients usually ask. Dietary guidance is the most often topic of interest. Proposal of daily meals are also suggested. It is very important for information to be well organized and easily accessed in order to achieve its value. Additionally, it is necessary for the information access to be secure and to give correct information to the right people at the right time to deliver the best possible care.

**(vii) Advertising Service**

In order to achieve sustainability of the system and funds for further development, an online advertising module is planned. The service also connects advertisers and marketers with physicians and other medical professionals and the patients as well. Promotions and advertising are an integral part of the health virtual community, and they have a great effect on human perception.

## 3. User Satisfaction

**Figure 4:**
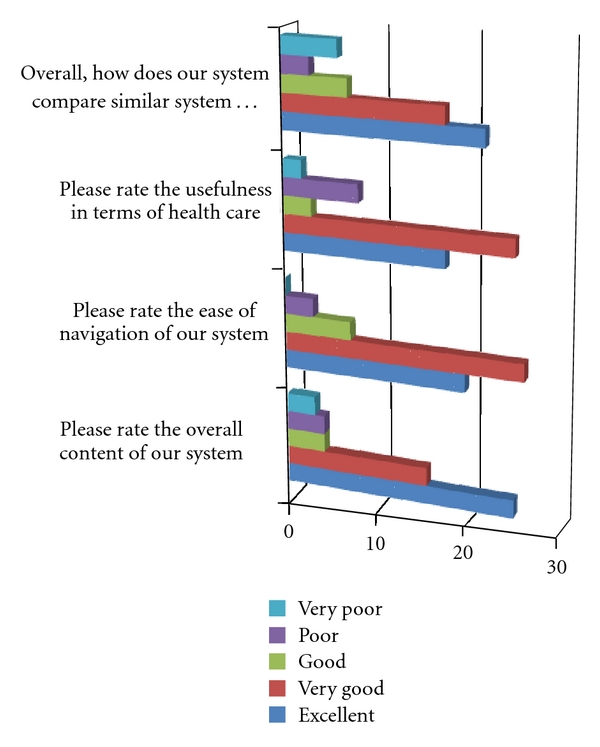
Ratings of the system, based on Table 1.

**Figure 5:**
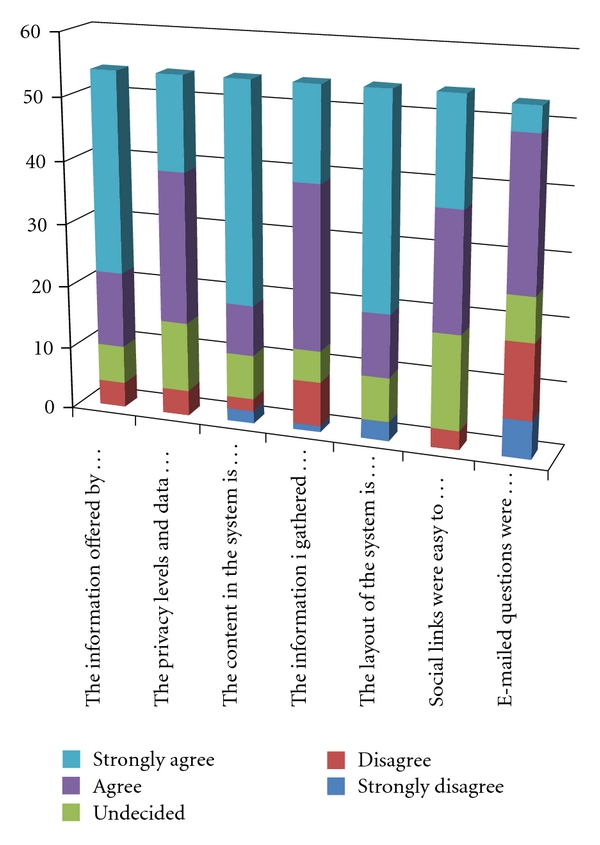
User evaluations, based on Table 2.

We have evaluated our system by providing a questionnaire to the initial user group that measured the satisfaction level of the users. The questionnaire contained 15 questions which were designed in a user-friendly format in order to accomplish easy competition by the respondents. The survey completed a 2-month-long period of trial use and evaluation of the system. The results and analysis can be grouped in the Figures 4 and 5. 

### 3.1. Response Rate

There were 54 respondents to our questionnaire. Respondents belong to different user profiles. A smaller portion: 15 of them were young and the remaining (34) were older diabetic patients. There were 5 medical specialists. Each one originated from different region of our country.

### 3.2. Usefulness of the Data Content of Our System

A graphical overview of the usefulness rating is visible in Figure 4. Most of the respondents were satisfied with the data content provided from our system. Around 66.6% of them have rated the overall content in our system as “Excellent” and even 27.7% as “Very Good”. Still, a few older participants have stated that they would rather go to a medical institution than use our platform.

### 3.3. Ease of Navigation

Over half the participants (59%) gave a rating “Very Good” and “Good” for the ease of navigation through the system. However, there were miscellaneous comments about navigation like “After I have registered the system and started using it, I fully understood how it is organized.”

### 3.4. System's Design

The System's design was evaluated as simple, creative, and systematic. Results have showed that the primary objectives to identify users' requirements and to build a system that satisfies these requirements were met.

### 3.5. Overall System Expectations

Nearly three-fourths (75%) of respondents indicated that the system and the information gathered met their expectations. Out of the remaining responders, 14% said “Disagree” and “Strongly Disagree” and 9% were undecided. Both negative and positive comments related to the user expectations were received. Some participants commented that they expected improvements to the system as an ongoing, continuous process. Others demanded improvements in navigation and online resources.

### 3.6. Comparing Our System with Similar Systems Used

Nearly 72% of respondents rated our system as “Excellent” and “Very Good” in comparison with other similar systems, and only 12% answered as “Very Poor” and “Poor”. Naturally, the users are mostly experienced with using generic social networks (Facebook, Myspace, Twitter), and the specifics of a healthcare social network were new to both the patients and developers. Comparative conclusions were constantly drawn and ideas implemented.

## 4. Future Trends

Although Internet access is increasing worldwide, there is still a limited pool of people who can use IT without difficulties, so an Internet-based diabetes care system must address and overcome its limitations before application. It is believed that the dramatic development of IT and its application to health management systems will have a crucial role [14]. Therefore, further adapting the user interface for users with eyesight problems (often in diabetic patients) or older users is crucial for our system. 

Integration of the virtual community with the public health care system would surely help its growth and stabilization. Adapting the functionalities and features for an international audience is the next step aimed at grouping a larger portion of patients exchanging experiences, due to the limitation that our small country poses. 

The web interface of the system is usable on mobile devices as well. However, it is our next goal to implement native applications for the most popular platforms (Android, iPhone). Such native applications would enhance user experience, decrease network traffic and above all, open possibilities for more direct telemedicine functionalities (GPS location transmission from a patient with acute crisis in need of help, transmission of other vital measurements—heart rate, blood pressure, video consultations on site, urgent transmission of lab results, and blood sugar measurements). 

Our future plans are to continue to deliver the high level of communication and services, extend the model platform with additional services that will provide facilities such as receiving messages on mobile devices, and chat rooms. The main goal is to constantly improve the quality and quantity of services.

## 5. Conclusion

The appeal of healthcare virtual communities transcends their ability to neither entertain connect people; they could also save lives. Virtual communities can be tools to promote health treatment strategies, change the patient-caregiver relationship, and to reform the way healthcare is delivered. In fact, they already have been used to sustain healthy lifestyle changes, encourage patients' dedication in their treatment, educate, and improve the access to needed information. The proposed diabetes healthcare virtual community model promotes highly effective services supported by major communication capabilities and easy information access. One of the major benefits of the healthcare social network is building online social connections with peer patients, exchange experiences, share problems, and commonly search for solutions. Chat rooms, group meetings, and consultations are all virtually brought in the patient's home, anytime, anywhere. With mobile devices, the provided services are available on the go. Medical personnel are present to verify data, strengthen trust in the system, and maintain the quality of the information provided.

## Figures and Tables

**Table 1:** Usefulness results.

	Excellent	Very good	Good	Poor	Very poor
Please rate the overall content of our system	24	15	4	4	3
Please rate the ease of navigation of our system	19	25	7	3	0
Please rate the usefulness in terms of health care	17	24	3	8	2
Overall, how does our system compare to similar systems you have used?	21	17	7	3	6

**Table 2:** System evaluation.

	Strongly disagree	Disagree	Undecided	Agree	Strongly agree
The information offered by the system is easy to understand	0	4	6	12	32
The privacy levels and data control is satisfactory	0	4	11	24	15
The content in the system is up-to-date	2	2	7	8	35
The information I gathered met my needs	1	7	5	26	15
The layout of the system is well organized and clear	3	0	7	10	34
Social links were easy to create and control	0	3	15	19	17
E-mailed questions were answered in a timely manner	6	12	7	24	4
